# Psychometric properties of ability to contribute measurements as a domain of functional ability of older persons: a COSMIN systematic review

**DOI:** 10.1093/ageing/afad099

**Published:** 2023-10-30

**Authors:** Luis Miguel Gutiérrez-Robledo, Pamela Tella-Vega, Rosa Estela García-Chanes, Luis Raymundo Lozano-Juárez, Raúl Hernán Medina-Campos, Salvador García-Andrade, Alberto Escamilla-Núñez, Jotheeswaran Amuthavalli Thiyagarajan, Theresa Diaz, Christopher Mikton, Carmen García-Peña

**Affiliations:** Instituto Nacional de Geriatría, Ciudad de México, México; Instituto Nacional de Geriatría, Ciudad de México, México; Instituto Nacional de Geriatría, Ciudad de México, México; Instituto Nacional de Geriatría, Ciudad de México, México; Global Health Research Departament, Juntendo University, Tokyo, Japan; Instituto Nacional de Geriatría, Ciudad de México, México; Geriatric deparment, Hospital General ISSSTE, Querétaro, México; Analysis and Synthesis of the Evidence Research Unit, National Medical Center XXI Century, Instituto Mexicano del Seguro Social, Mexico City, México; Department of Maternal, Newborn, Child, Adolescent Health and Ageing, World Health Organization, Geneva, Switzerland; Department of Maternal, Newborn, Child, Adolescent Health and Ageing, World Health Organization, Geneva, Switzerland; Department of Social Determinants of Health, Division of Healthier Populations, World Health Organization, Geneva, Switzerland; Instituto Nacional de Geriatría, Ciudad de México, México

**Keywords:** healthy ageing, functional ability, ability to contribute, older people, systematic review

## Abstract

**Background:**

Older person’s ability to contribute covers contributions divided into five subdomains: assisting friends and neighbours, mentoring peers and younger people, caring for family, engaging in the workforce and voluntary activity.

**Objective:**

To evaluate the psychometric properties of ability to contribute measurements as a domain of functional ability of older persons using Consensus-based Standards for the selection of health Measurement Instruments (COSMIN) methodology for systematic reviews.

**Methods:**

A systematic search was performed in PubMed, Embase and PsycINFO databases, for observational studies published within the last 10 years. The measurement properties of these ability measures were evaluated against the COSMIN taxonomy. Risk-of-bias assessment was performed using the COSMIN Risk of Bias checklist.

**Results:**

Of the 32,665 studies identified, we selected 19, of which the main purpose was to develop or validate an instrument or have related items that measure at least one of the subdomains. None of the instruments contained items that were fully related to the five subdomains, 60% (*n* = 12) were related to voluntary activities and 15% (*n* = 3) to mentoring peers and younger people. As for psychometric properties, two studies assessed content validity. Factor analysis was used to evaluate structural validity in 10 studies. Internal consistency was evaluated in 63% of the instruments and Cronbach’s alpha ranges from 0.63 to 0.92. No study reported predictive validity. A very limited overview of their scope and limitations for their application was observed.

**Conclusions:**

We found no single instrument measuring all subdomains of ability to contribute. We found several instruments containing items that could indirectly measure some of the subdomains of the ability to contribute.

## Key Points

- The ability of older persons to contribute to society is one of the five domains of functional ability.
- The ability to contribute is multifaceted and overlaps with several other attributes.
- No previous efforts have been made to systematically search the literature for instruments measuring the ability to contribute.

## Introduction

Improving older persons’ ability to contribute to family and society is key to promoting healthy ageing and sustainability. According to the perspective of healthy ageing, the ability to contribute is one of the domains of functional ability, and is defined as the capabilities that allow older persons to provide or supply things that are useful to, or valued by, the society [1, 2]. At the same time, the ability to contribute considers the rights, liberties and opportunities that older persons hold valuable. This ability covers a myriad of contributions that older people make to their families and communities, which are closely associated with engagement in social and cultural activities and represents efforts to measure their opportunities to contribute to different aspects of social life. Some contributions may overlap with other domains of functional ability such as the ability to build and maintain relationships and the ability to learn, grow and make decisions [2, 3]. To differentiate the ability to contribute from other domains of functional ability, the contributions of older people are divided into five subdomains proposed by the World Health Organization (WHO) [3]: (i) assisting friends and neighbours [4]; (ii) mentoring peers and younger people; (iii) caring for family members; (iv) being engaged in the workforce and (v) voluntary activity [2, 5]. Of note, remuneration is not definitory of a contribution. Older persons often do not receive monetary compensation for their participation in these activities, but a transactional nature is intrinsically acknowledged wherein both parties derive some form of benefit, hence the nature of contribution [6]. There is also a subjective component that implies that different persons may value different ways of contribution [4].

The United Nations Decade of Healthy Ageing (2021–23) calls for strengthening data and research on healthy ageing, including the measurement of older persons’ ability to contribute to society [3]. It is crucial to build greater national capacities to respond to the needs of ageing populations and to perform better monitoring of such response through age-disaggregated health data, including measures of functional ability and its domains [1]. There is a growing body of research on intrinsic capacity [7, 8], but there is still little research on the domains of functional ability. Specifically, there has not been an effort to systematically review instruments that measure the ability to contribute and their psychometric properties [5, 9].

This study aims to evaluate the psychometric measurement properties of ability to contribute measurements as a domain of functional ability of older persons through COSMIN methodology for systematic reviews.

## Method

This systematic review followed the Consensus-based Standards for the selection of health Measurement Instruments (COSMIN) methodology for conducting systematic reviews of psychometric properties [10] and the Preferred Reporting Items for Systematic Reviews and Meta-Analyses (PRISMA) statement [11]. The protocol was also registered in the PROSPERO international prospective register of systematic reviews, with the registration number CRD42022299500.

### Search strategy and selection criteria

We searched PubMed, Embase and PsycINFO to identify original research studies published between April 2012 and August 2022. The search strategy was developed using free text and controlled vocabulary (MeSH terms) and adapted for each database to identify articles describing the use of instruments that measure one, some or all subdomains of the ability to contribute and assessing their psychometric properties.

The search was restricted to observational studies of persons aged 60 years and older, published within the last 10 years in English. No restrictions were made as to the setting of the population. We included studies if they referred to at least one of the subdomains. Some of the search terms included for each subdomain are ‘gift giving’, ‘helping behaviour’ (assisting friends and neighbours), ‘mentoring’, ‘teaching’ (mentoring peers and younger people), ‘infant care’, ‘grandparents’ (caring for family members), ‘work’, ‘employment’ (being engaged in the workforce) and ‘volunteers’ (voluntary activity). The search also incorporates other terms related to the contribution of older persons (i.e. ‘social participation’ or ‘social environments’) and terms related to the psychometric properties of the instruments (i.e. ‘psychometrics’, ‘validation studies’) as defined by COSMIN. The complete search strategy can be reviewed in Supplementary Appendix 1.

### Quality assessment and data extraction

The results of each of the systematic searches carried out in the meta-bases PubMed/MEDLINE, Embase and PsycINFO were reviewed to detect all possible measures of interest. The review was carried out by three pairs of collaborators, over three stages:

- The titles and abstracts were randomly distributed in pairs and the articles were selected if they were related to any of the subdomains of the ability to contribute. The collaborators discussed the discrepancies and reached an agreement whether to include an article or not. The articles were classified in one of two categories: 1 = article selected for full review, 2 = rejected.
- Each pair separately reviewed the full-text articles that were selected and classified them as follows: (a) the main purpose of the study was to develop or validate an instrument that measured the ability to contribute or any of its subdomains; (b) the study used an instrument containing questions or items related to the ability to contribute or any of its subdomains; and (c) the article did not measure any of the subdomains of the ability to contribute. We decided to include articles classified as (a) and (b) in this review. For articles in category (b), the original reference was searched. Articles using dichotomous variables and classified as (c) were excluded.
- For selected articles (a and b), two summary tables were made to systematise and analyse the most relevant characteristics of the instruments for measuring the ability to contribute and their subdomains in an Excel spreadsheet: the internal ID number of the article, its title and its citation; the name of the instrument or instruments used; the items related to the ability to contribute, its description, the subdomain of the ability to contribute that measures, the type of variable, range or categories and comments for clarification. A third table was made describing the psychometric properties of the instruments or items, when available.

### Analysis and risk-of-bias assessment

A narrative synthesis was performed to characterise the studies included in the systematic review.

In order to analyse the scope and limitations of the instruments selected to measure the ability to contribute or its five subdomains, the following characteristics were described: instrument name, total items, number of items related to any of the subdomains, subdomain(s) measured and also we include the verb or the perspective used in the item because it could be different connotations can do, could do, be able to do among others.

Assessment of the measurement properties of the instruments was carried out using the COSMIN taxonomy of measurement properties and definitions for health-related patient-reported outcomes. Nine psychometric properties were assessed for each measure using the COSMIN Risk of Bias checklist [12]: (1) content validity, (2) structural validity, (3) internal consistency, (4) reliability, (5) invariance measurement, (6) criterion validity and (7) reliability. The COSMIN guidelines were used to evaluate the measurement properties of each scale in terms of four-point scale based on the study design and methodology (very good, adequate, doubtful and inadequate) [13, 14]. To assess this methodological quality score, for each psychometric property and according to COSMIN checklist, the lowest score was taken as a reference point (the lowest score counted as a principle). Two reviewers evaluated each article following the established points and disagreements were resolved by consensus.

Responsiveness was outside the scope of this review. The interpretability is not considered to be a psychometric property under the COSMIN framework and was therefore not described in this review.

## Results

### Search results

**Figure 1:**
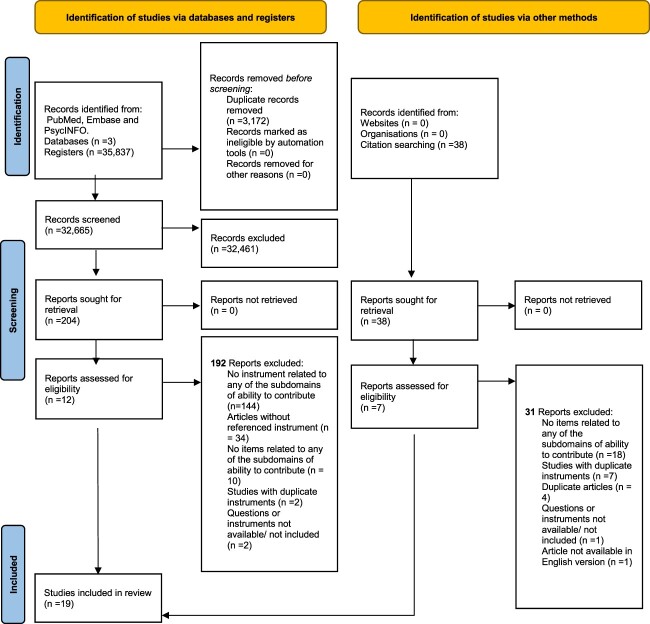
Flow chart of study selection. Source: Own elaboration based on the PRISMA method.

The detailed flow chart of study selection is described in Figure 1. The searches of the three meta-bases yielded 35,837 results. After removing duplicate records, a total of 32,665 records remained for screening. Of these, we selected 204 for full-text retrieval. We identified 38 other records from hand-searching the reference lists of these articles, for a total of 242 full-text articles. The review process performed in pairs excluded 223 articles, the most common reason being that the article was not related to the ability to contribute or any of its subdomains (*n* = 144). Other reasons for exclusion are described in Figure 1. Finally, we included 19 studies in the systematic review. The search was originally restricted only to the group of people over 60 years of age, but because some articles were found related to the concept even in other groups of age, it was decided to keep those that did not meet the established age cut-off point. The full list of studies can be reviewed in Supplementary Appendix 2.

### Characteristics of studies

The 19 studies included in the review were geographically diverse and included 15 countries, of which Canada, Japan, Netherlands and the USA were the countries with the highest frequency (three studies per country) (Table 1).

Sample size among the studies was heterogeneous, ranging from 67 to 123,760, with 57.9% of the studies having samples of 500 people or fewer. Only 31.6% (*n* = 6) of the studies included exclusively older persons over 60 years, 21% (*n* = 4) included population over 50 years of age, 31% (*n* = 6) included population under 50 years of age and 15.8% (*n* = 3) did not report the age of their participants. Women represented more than 50% of the sample in 12 of the 19 studies, and 3 studies did not report the sex of their participants.

Most studies were carried out in the community (78.9%). National surveys were used in four studies: 2013 Survey of the Shanghai Elderly Life and Opinion [15]; Health and Retirement Study [16]; Japan Gerontological Evaluation Study [17]; NIDI Pension Panel Study [18] (Table 1).

### Approach to subdomain measurements of the ability to contribute

When analysing the instruments developed or used in the studies in relation to the way in which they could be capturing and measuring the ability to contribute or any of its domains, we did not find instruments specifically designed to operationalise this concept. However, we identified some instruments that contained items that could be related to some of the attributes of the concept and to some of its subdomains.

**Table 1:** Characteristics of instruments included

Citation	Country	Population characteristics				Characteristics of the instrument								
		*N*	Age (mean, range)	Gender, female (%)	Setting	Name	Type of item^a^	Items related with any subdomain/ total items	Number of items linked to each subdomain of the ability to contribute^b^ (*N*)					Aspect that measure
									A	B	C	D	E	
McAdams & Logan, 1993 [20]	USA	152	NR, 22–72	52.6	Community	Loyola Generativity Scale	L	3/20		2			1	Try to
Noreau *et al*., 2004 [21]	Canada	84	78, NR	73	Community and institutionalised	The Life H-Short version 3.1	C	3/69			1	1	1	To do
de Zwart *et al*., 2002 [22]	Netherlands	97	51, 40–60	1.03	Employees of construction industry	Work Ability Index	L	2/7				2		Able to
Wilkie *et al*., 2005 [23]	UK	1,117	NR, 50+	NR	Community	Keele Assessment of Participation	L	3/11	1	1		1		To do
Richard *et al*., 2009 [24]	Canada	282	72, 58–92	74	Community	Social Participation scale	F	2/10	1				1	To do
Ghaziani *et al*., 2013 [25]	Denmark	67	54, 22–74	52	Clinical	IPAQ	L	3/32	1^d^		1^d^	2		To do
Stevelink *et al*., 2012 [26]	Bangladesh, India, Indonesia, Nepal, Brazil and Netherlands	5,125	44, NR	43	Community	Participation Scale (P-Scale)	C	3/18				3		To do as your peers
Verduin *et al*., 2014 [27]	Rwanda	393	38.5 (men), 36.1 (women), 16–81	54	Community	Byumba Social Functioning Questionnaire (BSFQ)	L	3/20			2	1		Degree of difficulty completing
Resnick *et al*., 2013 [19]	USA	127	88, NR	78	Community	Engagement in volunteer activities	D	27/27					27	To do/would you be interested
Hirano *et al*., 2015 [28]	Japan	110	81, 65–85+	100	Community	Social Activities Scale for Older Women Requiring Support	L	3/42	3					To do
Lovell *et al*., 2015 [29]	New Zealand	306	NR, 18–71+	55	Community	Community Capacity Instrument	L	2/46					2	Can do
Zhang *et al*., 2015 [15]	China	3,418	NR, 50–60+	52	Community^c^	Productivity Activity	D	3 Not scale			3			To do
Infurna *et al*., 2016 [16]	USA	13,262	71, 60+	58	Community^c^	Volunteering (only question)	D	1 Not scale					1	To do
Hong *et al*., 2017 [30]	Canada	352	NR, 20–60	65.6	Workers from a government agency in Quebec	Work Role Functioning Questionnaire	L	1/27				1		Feel you have done
Saito *et al*., 2017 [17]	Japan	123,760 senior citizens; 702 communities	NR, 65+	NR	Community^c^	Social Capital Scale	F/L	3/14	1	1			1	To do
Nikkhah *et al*., 2018 [18]	Iran	500	NR, 60–90	33	Community^c^	OPQOL	L	1/35			1			To do
Grünwarld *et al*., 2021 [31]	Netherlands	4,882	NR, 60–65	NR	Community	Informal caregiving, formal volunteering, and grandparenting (only questions)	C	3 Not scale			2		1	To do
Oki & Tadaka, 2021 [32]	Japan	790	74, 65–93	1	Community	Social Contact Self-Efficacy Scale for Third Agers (SET)	L	2/18	1				1	Able to
Chen & Zhang, 2021 [33]	China	1,458	NR, 50–80+	57	Community	Community Participation Survey	L	5/5					5	To do

^a^Type of item: (L = Likert, C = Categories, D = Dichotomous, F=Frequency).

^b^A = Assisting friends and neighbours, B = Mentoring peers and younger people, C = Caring for family member, D = Being engaged in the workforce, E = Voluntary activity.

^c^Used ongoing national surveys.

^d^Same item.

In Table 1, we summarise the main characteristics of the items of 16 instruments and three single questions related to the ability to contribute. Most instruments contained one to three items related to the ability to contribute. Engagement in voluntary activities [19] was the instrument with the highest number of related items (*n* = 27), but most of them were related only to a list of different types of voluntary activities. None of the instruments contained items related to the five subdomains; 60% of them (*n* = 12) contained items related to voluntary activities and only 15% (*n* = 3) contained items related to mentoring peers and younger people.

From the 73 items connected to ability to contribute, more than half are related to the subdomain ‘Voluntary activity’ (61.6%, *n* = 45); 17.8% (*n* = 13) to ‘Being engaged in the workforce’, 11% (*n* = 8) to ‘Caring for family members’, 11% (*n* = 8) to ‘Assisting Friends and neighbours’ and 5.5% (*n* = 4) to ‘Mentoring peers and younger people’. Five items (6.8%) were found to be related to two subdomains simultaneously: two items from the Impact on Participation and Autonomy Questionnaire (IPAQ) [25], one from the Keele Assessment of Participation [23] and one from the Older People’s Quality Of Life questionnaire (OPQOL) [18] were related to both paid and voluntary work; while one item from the IPAQ [25] was related to both ‘Assisting friends and neighbours’ and ‘Caring for family members’.

Concerning the subdomain ‘Voluntary activity’, we identified items related to the skills or possibilities to perform such activities (‘I can reach out to person in need on the street’ [32] or ‘I am quick to work with others when I see a need within the community’, ‘I support the local school wherever I can’) [29] and items that focus on whether they perform such activity (‘I do volunteer to work for a charity’ [20], ‘Are you involved in volunteer work?’) [31]. It is important to consider that some items specify the type of activities.

Regarding the subdomain ‘Being engaged in the workforce’, we found items that capture whether this paid activity is carried out, its importance and the way in which it is done, such as ‘Keeping a paid job’ [21], ‘I get/keep a paid job, I do paid or unpaid work or activities that give me a role in life’ [18]. Other instruments like Work Ability Index [22] measure current job skills with respect to current demands (‘work ability in relation to the demands of the job’) and about the past (‘current work ability compared with the lifetime best’). An item was found related to how you feel about what you can do (‘Feel you have done what you are capable of doing’), which was identified in the Work Role Functioning Questionnaire [30]. Also, two items were identified in the Participation Scale (‘Do you work as hard as your peers do?’, ‘Do you contribute to the household economically in a similar way to your peers?’) that measure ability through comparison with peers.

In relation to the subdomain ‘Caring for family members’, items are related to participation: ‘Do you provide help to relatives or friends who are sick or who need it?’ ‘Do you ever take care of your grandchildren?’ [31], ‘Care assistance provided to their grandchildren’ [15], the importance of the activity ‘Carrying out family or domestic tasks as the main participation’ [21] and the difficulties that may arise to perform them [27].

In relation to the subdomain ‘Assisting Friends and neighbours’, the related items focus on the ability and possibilities to do so: ‘I am able to support each other with my family and others in times of need’ [32], ‘Do you listen to someone’s concerns and complaints?’ [17], ‘Talk with the people in the neighbourhood, check on condition of each other’ [28], ‘Help or support others. My chance of helping or supporting people in any way are’ [25].

Concerning the subdomain ‘Mentoring peers and younger people’, related items are less frequent: ‘How often do you attend activities to teach skills or pass on experiences to others?’ [17] and the items ‘I try to pass along the knowledge I have gained through my experiences’ and ‘I have important skills I try to teach others’ were identified in the Loyola Generativity Scale [20].

### Summarised of findings and quality of evidence

We assessed the psychometric properties of the instruments and items selected for this review and summarised our findings in Table 2.

**Table 2:** Psychometric properties and best evidence synthesis of outcome measures against the COSMIN risk-of-bias checklist rating and level of evidence for the measurement property [13, 14]

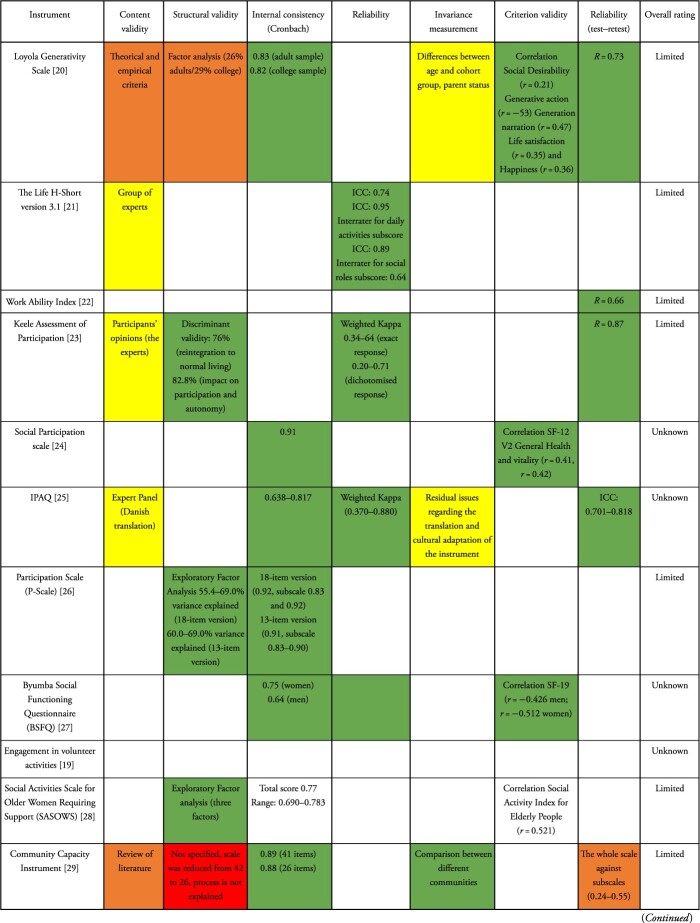
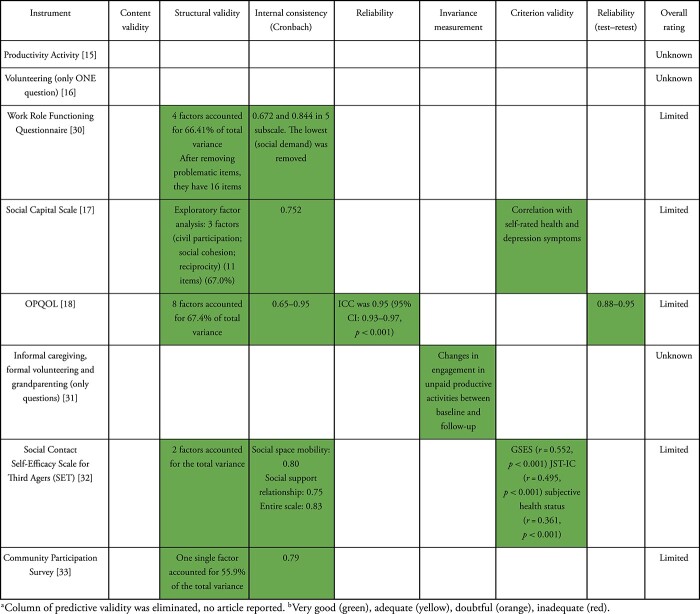

### Content validity

From the 19 studies, only two included the strategy of group of experts [21, 23] on the comprehensiveness component. One article [29] included a review of the literature and another used theorical and empirical criteria [20]. It should be noted that in the case of the translation of an instrument, an expert panel was used [25]. In total, only five studies showed this validation (26.3%).

### Structural validity

Altogether, 10 of the 19 articles assessed the structural validity (52.6%). Exploratory factor analysis was used in seven articles to identify dimensions (% total variance) and define the number of elements for the instruments [17, 18, 26, 28, 30, 32, 33]. The articles that show greater explained variability are OPQOL [18], Social Capital Scale [17], Work Role Functioning Questionnaire [30] and *P-*Scale [26]. Discriminant validity was used in only one study [23]. The results of three studies were methodologically of low quality [28, 29, 32].

### Internal consistency

Most of the studies included the Cronbach’s alpha criterion (63.2%) and showed values >0.70, ranging from 0.638 to 0.92. Specifically, in some studies, this indicator was estimated for different versions of the instrument [26, 29], subscales or subdomains of instruments [30, 32], or subsamples [20, 27]. The overall rating was sufficient and of high quality for the internal consistency.

### Reliability

Reliability was reported for four studies (21.2%). Three studies [18, 21, 25] evaluated the intraclass correlation coefficient (ICC) for reliability, while the results were insufficient for the remaining studies.

### Predictive validity

None of the studies presented where instruments are validated present predictive validation.

### Measurement invariance

The measurement in different populations was analysed (21.2%) for four studies. According to the interest and objective of the instruments, various proposals were done to test differences between interest groups in the behaviour of the measurement; differences by age-cohort and parent status were analysed in the case of generativity [20], different communities for instruments that measure community capacity [29] and differences between activities and over time [31]. Only in the case of IPAQ (Danish version), this study showed limitations for this instrument in cultural adaptation [25].

### Criterion validity

Criterion validity was reported for six studies (31.6%). Correlation analysis was performed with other related measurements such as Social Desirability, Life satisfaction and happiness [20]; SF-12 [23]; SF-19 [27]; Social Activity Index for Elderly People [28]; Self-rated health and depression symptoms [17]; and GSES, JST-IC and subjective status [32]. In general, the range of correlation coefficients was low (0.21–0.55).

### Retest reliability

Retest reliability was reported for six studies (31.6%), ranging from 0.66 to 0.95, and only in one study it was evaluated by subscale [29].

Not all measures were available for every instrument or item, and five instruments had no psychometric properties reported at all. In general, when evaluating the psychometric properties of the selected instruments, a very limited overview of their scope and limitations for their application was observed. Internal consistency and structural validity were the most often validated properties regarding any of the subdomains of the ability to contribute, which suggests that they are being evaluated in their theoretical construct due to their complexity, since they involve psychological, social and cultural factors. The scarcity of information may also reflect the difficulties involved in capturing the heterogeneity of activities that can vary with respect to age, sex, place of residence and socioeconomic context.

## Discussion

The purpose of this review was to review the existing instruments aimed at measuring the ability to contribute and the psychometric properties of these. The results show that no specific instrument has been developed that overall measures the ability of older person to contribute, although some instruments contain items with a limited perspective that could be considered to measure this construct.

Healthy ageing is a relatively recent concept that is still being revised and refined. In the same fashion, functional ability and its subdomains are still not fully understood. Functional ability had been previously defined as the ability to perform activities of daily living without pain and fatigue, and had been closely related to physical fitness as a requisite for living independently [34]. However, the WHO framework of healthy ageing expands the scope of the concept beyond independent living and places emphasis on the subjective features that allow older persons to be and to do what they have reason to value. Meeting basic needs; learning, growing and making decisions; being mobile; building and maintaining relationships; and contributing to society are all part of the human experience, although different persons might prioritise them differently at different stages throughout their life. This subjectivity that is inherent to the subdomains of functional ability makes it particularly difficult to measure. It is therefore not surprising that so far only a few indicators of the ability to meet basic needs—particularly getting dressed, taking medications and managing money—are widely available from national surveys of member states [3]. The ability to contribute to society is, if anything, the most subjective of all five subdomains of functional ability, and possibly the most subjected to cross-cultural variability. This might explain why no specific measurement instrument for the ability to contribute to society was found in this review.

As with other domains of functional ability, the ability of older persons to contribute to society is a function of intrinsic capacity, the environment and the interactions between them. Any attempt to measure the ability to contribute must therefore consider these interactions and use properly phrased items. We found in this review that the wording is crucial because it might change the intention or the accuracy of the response. For example, some instruments focus on things that persons do that might represent a contribution to society, while others focus on what persons try to do, are able to do, can do or feel that they have done. This wide variability might result in similar items eliciting very different responses.

Noticeably, most of the studies included in this review contained social participation or participation as a key term. Social participation has been previously defined as ‘a person’s involvement in activities that provide interaction with others in society or the community’ [35]. This definition requires some level of involvement, engagement of performance with others and often involves a purpose of helping or benefiting others in some way. Accordingly, contributing to society is considered the highest or most complex form of social participation.

In our review, we found that items investigating the ability to contribute mostly referred to being engaged in the workforce or voluntary activity or assisting friends and neighbours. Whether this is a result of such subdomains being more common or having more evident beneficiaries or some other reason is unclear. On the contrary, mentoring peers and younger people was the least explored subdomain of the ability to contribute. In the case of being engaged in the workforce, some items enabled showing the way in which older people feel capable of responding to current work demands and in relation to what happened before. However, it is necessary to consider that this subdomain may be biased for gender reasons, considering that workforce participation is higher in men, so it would be necessary to define this subdomain and complement with unpaid work that is also performed.

To our knowledge, no previous efforts have been made to systematically search the literature for instruments or items measuring the ability of older persons to contribute to society. Measuring this domain of functional ability is critical not only to improve our understanding of the concept, but to ensure that older persons are valued and to combat ageism, as stated by WHO in the Global strategy and action plan on ageing and health [36].

The most evident limitation in this review is the absence of instruments that measure the ability to contribute in a wholesome manner. For this reason, we decided to search for instruments that partially measured one or more of the subdomains of the ability to contribute. The items measuring these subdomains might be a starting point for the development of a dedicated instrument to measure the ability to contribute. Should this course of action be selected, it must be noted that some clarifications are still necessary. For instance, it is crucial to determine whether the ability to contribute should be measured in terms of the specific outcomes of the contribution—i.e. how many hours a week an older person dedicates to spends doing voluntary work—or in terms of the barriers or facilitators they encounter when trying to contribute—i.e. whether an older person finds the proper means to perform such voluntary work. While both types of items might confer relevant information, the latter might be harder to develop in a way that can be adapted to different settings.

Another relevant finding is that the ability to contribute is multifaceted and overlaps with several other attributes. We identify concepts such as social functioning, self-efficacy, generativity, community capacity, work ability and resilience, which can be related to the ability to contribute. They capture together the individual and social skills and abilities of individuals to perform in different spaces and situations. In addition, the instruments related to these concepts can be used as a reference to assess the validity of the instrument that measure the ability to contribute that will be developed later.

A relevant matter is whether the ability to contribute can or should be measured based solely on individual assessments, given the strong influence of environmental factors. The ability to take part in formal employment is a good example of such influence. While older persons in high-income countries might find it satisfying and meaningful to be able to continue to work past the retirement age, those in low-income countries might not have a choice but to continue to work into their old age due to lack of other means of survival. When asked whether they continue to participate in the workforce, older persons from high- and low-income countries might respond similarly but for entirely different reasons and influenced by completely different factors. Thus, individual assessments might not be sufficient, and a more systemic approach might be needed to measure the environmental factors influencing older persons’ ability to contribute to their society.

## Conclusion

We found no instruments aimed at measuring the ability of older persons to contribute to society. However, we found several instruments containing items related to one or more subdomains of the ability to contribute, namely, being engaged in the workforce, voluntary activity, assisting friends and neighbours, caring for family members and mentoring peers and younger people. There is significant overlap with the concept of social participation, which requires further clarification for the purpose of properly measuring the ability to contribute.

## Supplementary Material

aa-23-0316-File003_afad099

aa-23-0316-File004_afad099
