# Supplementary material for: Psychometric properties of ability to contribute measurements as a domain of functional ability of older persons: a COSMIN systematic review
Source: Age Ageing. 2023 Oct 30;52(Suppl 4):iv138–48. doi: 10.1093/ageing/afad099 (PMC10615053; doi:10.1093/ageing/afad099)
Supplement: aa-23-0316-File003_afad099 [file aa-23-0316-file003_afad099.docx]

| World Health Organization: *Measurement of Healthy Ageing.*  Psychometric properties of ability to contribute measurements as a domain of functional ability of older persons: a COSMIN systematic review.  **SUPPLEMENTARY DATA**  **Appendix 1.** Search Terms | | | |
| --- | --- | --- | --- |
| **Search number** | **Query** | **Filters** | **Results** |
| **14** | #10 NOT #11 | **Aged: 65+ years, from 2012 - 2022** | **21,208** |
| **13** | #10 NOT #11 | **from 2012 - 2022** | **21,702** |
| **12** | #10 NOT #11 |  | **41,637** |
| **11** | (‘delphi-technique’[ti] OR cross-sectional[ti] OR "biography"[Publication Type] OR "case reports"[Publication Type] OR "comment"[Publication Type] OR "directory"[Publication Type] OR "editorial"[Publication Type] OR "festschrift"[Publication Type] OR "interview"[Publication Type] OR "legislation"[Publication Type] OR "letter"[Publication Type] OR "news"[Publication Type] OR "newspaper article"[Publication Type] OR "patient education handout"[Publication Type] OR "consensus development conference"[Publication Type] OR "consensus development conference, nih"[Publication Type] OR "practice guideline"[Publication Type]) NOT ("animals"[MeSH Terms] NOT "humans"[MeSH Terms]) |  | **4,472,349** |
| **10** | #8 AND #9 |  | **43,271** |
| **9** | ((instrumentation[sh] OR methods[sh] OR "Comparative Study"[pt] OR "psychometrics"[MeSH] OR psychometr*[tiab] OR clinimetr*[tw] OR clinometr*[tw] OR "outcome assessment"[tiab] OR "outcome measure*"[tw] OR "observer variation"[MeSH] OR "observer variation"[tiab] OR "Health Status Indicators"[Mesh] OR "reproducibility of results"[MeSH] OR reproducib*[tiab] OR "discriminant analysis"[MeSH] OR reliab*[tiab] OR unreliab*[tiab] OR valid*[tiab] OR "coefficient of variation"[tiab] OR coefficient[tiab] OR homogeneity[tiab] OR homogeneous[tiab] OR "internal consistency"[tiab] OR (cronbach*[tiab] AND (alpha[tiab] OR alphas[tiab])) OR (item[tiab] AND (correlation*[tiab] OR selection*[tiab] OR reduction*[tiab])) OR agreement[tw] OR precision[tw] OR imprecision[tw] OR "precise values"[tw] OR test-retest[tiab] OR (test[tiab] AND retest[tiab]) OR (reliab*[tiab] AND (test[tiab] OR retest[tiab])) OR stability[tiab] OR interrater[tiab] OR inter-rater[tiab] OR intrarater[tiab] OR intra-rater[tiab] OR intertester[tiab] OR inter-tester[tiab] OR intratester[tiab] OR intra-tester[tiab] OR interobserver[tiab] OR inter-observer[tiab] OR intraobserver[tiab] OR intra-observer[tiab] OR intertechnician[tiab] OR inter-technician[tiab] OR intratechnician[tiab] OR intra-technician[tiab] OR interexaminer[tiab] OR inter-examiner[tiab] OR intraexaminer[tiab] OR intra-examiner[tiab] OR interassay[tiab] OR inter-assay[tiab] OR intraassay[tiab] OR intra-assay[tiab] OR interindividual[tiab] OR inter-individual[tiab] OR intraindividual[tiab] OR intra-individual[tiab] OR interparticipant[tiab] OR inter-participant[tiab] OR intraparticipant[tiab] OR intra-participant[tiab] OR kappa[tiab] OR kappa's[tiab] OR kappas[tiab] OR repeatab*[tw] OR ((replicab*[tw] OR repeated[tw]) AND (measure[tw] OR measures[tw] OR findings[tw] OR result[tw] OR results[tw] OR test[tw] OR tests[tw])) OR generaliza*[tiab] OR generalisa*[tiab] OR concordance[tiab] OR (intraclass[tiab] AND correlation*[tiab]) OR discriminative[tiab] OR "known group"[tiab] OR "factor analysis"[tiab] OR "factor analyses"[tiab] OR "factor structure"[tiab] OR "factor structures"[tiab] OR dimension*[tiab] OR subscale*[tiab] OR (multitrait[tiab] AND scaling[tiab] AND (analysis[tiab] OR analyses[tiab])) OR "item discriminant"[tiab] OR "interscale correlation*"[tiab] OR error[tiab] OR errors[tiab] OR "individual variability"[tiab] OR "interval variability"[tiab] OR "rate variability"[tiab] OR (variability[tiab] AND (analysis[tiab] OR values[tiab])) OR (uncertainty[tiab] AND (measurement[tiab] OR measuring[tiab])) OR "standard error of measurement"[tiab] OR sensitiv*[tiab] OR responsive*[tiab] OR (limit[tiab] AND detection[tiab]) OR "minimal detectable concentration"[tiab] OR interpretab*[tiab] OR ((minimal[tiab] OR minimally[tiab] OR clinical[tiab] OR clinically[tiab]) AND (important[tiab] OR significant[tiab] OR detectable[tiab]) AND (change[tiab] OR difference[tiab])) OR (small*[tiab] AND (real[tiab] OR detectable[tiab]) AND (change[tiab] OR difference[tiab])) OR "meaningful change"[tiab] OR "ceiling effect"[tiab] OR "floor effect"[tiab] OR "Item response model"[tiab] OR IRT[tiab] OR Rasch[tiab] OR "Differential item functioning"[tiab] OR DIF[tiab] OR "computer adaptive testing"[tiab] OR "item bank"[tiab] OR "cross-cultural equivalence"[tiab])) OR (Surveys and Questionnaires[MeSH Terms]) |  | **10,512,420** |
| **8** | #7 AND #6 |  | **82,128** |
| **7** | #1 OR #2 OR #3 OR #4 OR #5 |  | **709,834** |
| **6** | ((((aged[MeSH Terms]) OR (Grandparents[MeSH Terms])) OR (Aged, 65 and over[MeSH Terms])) OR (aging[MeSH Terms])) OR (Healthy Aging[MeSH Terms]) |  | **3,558,007** |
| **5** | (((((((volunteers[MeSH Terms]) OR (social participation[MeSH Terms])) OR (community participation[MeSH Terms])) OR (social behavior[MeSH Terms])) OR (social environment[MeSH Terms])) OR (Psychology, Developmental[MeSH Terms])) OR (Intergenerational Relations[MeSH Terms])) OR (social interaction[MeSH Terms]) |  | **465,054** |
| **4** | (((work[MeSH Terms]) OR (employment[MeSH Terms])) OR (Work engagement[MeSH Terms])) OR (workload[MeSH Terms]) |  | **153,492** |
| **3** | infant care[MeSH Terms] |  | **15,417** |
| **2** | ((mentoring[MeSH Terms]) OR (teaching[MeSH Terms])) OR (mentors[MeSH Terms]) |  | **103,728** |
| **1** | ((((giftgiving[MeSH Terms]) OR (Helping Behavior[MeSH Terms])) OR (coopérative behavior[MeSH Terms])) OR (custodial care[MeSH Terms])) OR (community networks[MeSH Terms]) |  | **56,647** |

| **Search number** | **Search detail** | **Filters** | **Results** |
| --- | --- | --- | --- |
| **14** | ((("gift giving"[MeSH Terms] OR "helping behavior"[MeSH Terms] OR "cooperative behavior"[MeSH Terms] OR "custodial care"[MeSH Terms] OR "community networks"[MeSH Terms] OR ("mentoring"[MeSH Terms] OR "teaching"[MeSH Terms] OR "mentors"[MeSH Terms]) OR "infant care"[MeSH Terms] OR ("work"[MeSH Terms] OR "employment"[MeSH Terms] OR "work engagement"[MeSH Terms] OR "workload"[MeSH Terms]) OR ("volunteers"[MeSH Terms] OR "social participation"[MeSH Terms] OR "community participation"[MeSH Terms] OR "social behavior"[MeSH Terms] OR "social environment"[MeSH Terms] OR "psychology, developmental"[MeSH Terms] OR "intergenerational relations"[MeSH Terms] OR "social interaction"[MeSH Terms])) AND ("aged"[MeSH Terms] OR "grandparents"[MeSH Terms] OR "aged"[MeSH Terms] OR "aging"[MeSH Terms] OR "healthy aging"[MeSH Terms]) AND ("instrumentation"[MeSH Subheading] OR "methods"[MeSH Subheading] OR "Comparative Study"[Publication Type] OR "psychometrics"[MeSH Terms] OR "psychometr*"[Title/Abstract] OR "clinimetr*"[Text Word] OR "clinometr*"[Text Word] OR "outcome assessment"[Title/Abstract] OR "outcome measure*"[Text Word] OR "observer variation"[MeSH Terms] OR "observer variation"[Title/Abstract] OR "Health Status Indicators"[MeSH Terms] OR "reproducibility of results"[MeSH Terms] OR "reproducib*"[Title/Abstract] OR "discriminant analysis"[MeSH Terms] OR "reliab*"[Title/Abstract] OR "unreliab*"[Title/Abstract] OR "valid*"[Title/Abstract] OR "coefficient of variation"[Title/Abstract] OR "coefficient"[Title/Abstract] OR "homogeneity"[Title/Abstract] OR "homogeneous"[Title/Abstract] OR "internal consistency"[Title/Abstract] OR ("cronbach*"[Title/Abstract] AND ("alpha"[Title/Abstract] OR "alphas"[Title/Abstract])) OR ("item"[Title/Abstract] AND ("correlation*"[Title/Abstract] OR "selection*"[Title/Abstract] OR "reduction*"[Title/Abstract])) OR "agreement"[Text Word] OR "precision"[Text Word] OR "imprecision"[Text Word] OR "precise values"[Text Word] OR "test-retest"[Title/Abstract] OR ("test"[Title/Abstract] AND "retest"[Title/Abstract]) OR ("reliab*"[Title/Abstract] AND ("test"[Title/Abstract] OR "retest"[Title/Abstract])) OR "stability"[Title/Abstract] OR "interrater"[Title/Abstract] OR "inter-rater"[Title/Abstract] OR "intrarater"[Title/Abstract] OR "intra-rater"[Title/Abstract] OR "intertester"[Title/Abstract] OR "inter-tester"[Title/Abstract] OR "intratester"[Title/Abstract] OR "intra-tester"[Title/Abstract] OR "interobserver"[Title/Abstract] OR "inter-observer"[Title/Abstract] OR "intraobserver"[Title/Abstract] OR "intra-observer"[Title/Abstract] OR "intertechnician"[Title/Abstract] OR "inter-technician"[Title/Abstract] OR "intratechnician"[Title/Abstract] OR "intra-technician"[Title/Abstract] OR "interexaminer"[Title/Abstract] OR "inter-examiner"[Title/Abstract] OR "intraexaminer"[Title/Abstract] OR "intra-examiner"[Title/Abstract] OR "interassay"[Title/Abstract] OR "inter-assay"[Title/Abstract] OR "intraassay"[Title/Abstract] OR "intra-assay"[Title/Abstract] OR "interindividual"[Title/Abstract] OR "inter-individual"[Title/Abstract] OR "intraindividual"[Title/Abstract] OR "intra-individual"[Title/Abstract] OR "interparticipant"[Title/Abstract] OR "inter-participant"[Title/Abstract] OR "intraparticipant"[Title/Abstract] OR "intra-participant"[Title/Abstract] OR "kappa"[Title/Abstract] OR "kappa's"[Title/Abstract] OR "kappas"[Title/Abstract] OR "repeatab*"[Text Word] OR (("replicab*"[Text Word] OR "repeated"[Text Word]) AND ("measure"[Text Word] OR "measures"[Text Word] OR "findings"[Text Word] OR "result"[Text Word] OR "results"[Text Word] OR "test"[Text Word] OR "tests"[Text Word])) OR "generaliza*"[Title/Abstract] OR "generalisa*"[Title/Abstract] OR "concordance"[Title/Abstract] OR ("intraclass"[Title/Abstract] AND "correlation*"[Title/Abstract]) OR "discriminative"[Title/Abstract] OR "known group"[Title/Abstract] OR "factor analysis"[Title/Abstract] OR "factor analyses"[Title/Abstract] OR "factor structure"[Title/Abstract] OR "factor structures"[Title/Abstract] OR "dimension*"[Title/Abstract] OR "subscale*"[Title/Abstract] OR ("multitrait"[Title/Abstract] AND "scaling"[Title/Abstract] AND ("analysis"[Title/Abstract] OR "analyses"[Title/Abstract])) OR "item discriminant"[Title/Abstract] OR "interscale correlation*"[Title/Abstract] OR "error"[Title/Abstract] OR "errors"[Title/Abstract] OR "individual variability"[Title/Abstract] OR "interval variability"[Title/Abstract] OR "rate variability"[Title/Abstract] OR ("variability"[Title/Abstract] AND ("analysis"[Title/Abstract] OR "values"[Title/Abstract])) OR ("uncertainty"[Title/Abstract] AND ("measurement"[Title/Abstract] OR "measuring"[Title/Abstract])) OR "standard error of measurement"[Title/Abstract] OR "sensitiv*"[Title/Abstract] OR "responsive*"[Title/Abstract] OR ("limit"[Title/Abstract] AND "detection"[Title/Abstract]) OR "minimal detectable concentration"[Title/Abstract] OR "interpretab*"[Title/Abstract] OR (("minimal"[Title/Abstract] OR "minimally"[Title/Abstract] OR "clinical"[Title/Abstract] OR "clinically"[Title/Abstract]) AND ("important"[Title/Abstract] OR "significant"[Title/Abstract] OR "detectable"[Title/Abstract]) AND ("change"[Title/Abstract] OR "difference"[Title/Abstract])) OR ("small*"[Title/Abstract] AND ("real"[Title/Abstract] OR "detectable"[Title/Abstract]) AND ("change"[Title/Abstract] OR "difference"[Title/Abstract])) OR "meaningful change"[Title/Abstract] OR "ceiling effect"[Title/Abstract] OR "floor effect"[Title/Abstract] OR "Item response model"[Title/Abstract] OR "IRT"[Title/Abstract] OR "Rasch"[Title/Abstract] OR "Differential item functioning"[Title/Abstract] OR "DIF"[Title/Abstract] OR "computer adaptive testing"[Title/Abstract] OR "item bank"[Title/Abstract] OR "cross-cultural equivalence"[Title/Abstract] OR "surveys and questionnaires"[MeSH Terms])) NOT (("delphi technique"[Title] OR "cross-sectional"[Title] OR "biography"[Publication Type] OR "case reports"[Publication Type] OR "comment"[Publication Type] OR "directory"[Publication Type] OR "editorial"[Publication Type] OR "festschrift"[Publication Type] OR "interview"[Publication Type] OR "legislation"[Publication Type] OR "letter"[Publication Type] OR "news"[Publication Type] OR "newspaper article"[Publication Type] OR "patient education handout"[Publication Type] OR "consensus development conference"[Publication Type] OR "consensus development conference, nih"[Publication Type] OR "practice guideline"[Publication Type]) NOT ("animals"[MeSH Terms] NOT "humans"[MeSH Terms]))) AND ((aged[Filter]) AND (2012:2022[pdat])) | **Aged: 65+ years, from 2012 - 2022** | **21,208** |
